# Supplementary material for: Deactivation and Regeneration of Nitrogen Doped Carbon Catalyst for Acetylene Hydrochlorination
Source: Molecules. 2023 Jan 18;28(3):956. doi: 10.3390/molecules28030956 (PMC9919228; doi:10.3390/molecules28030956)
Supplement: Supplementary file 1 [file molecules-28-00956-s001.zip › molecules-2152665-supplementary.pdf]

Supporting Information

Deactivation and Regeneration of Nitrogen Doped Carbon Catalyst for Acetylene Hydrochlorination

Fangjie Lu [1,2], Qinqin Wang [1,*], Mingyuan Zhu [1,2,*] and Bin Dai [1]

[1] School of Chemistry and Chemical Engineering, Shihezi University, Shihezi, Xinjiang 832003, China
[2] College of Chemistry and Chemical Engineering, Yantai University, Shandong 264010, China

Analytical criteria

The following Eq.S1 was used to calculate the conversion frequency (TOF) of acetylene as criteria for catalytic performance.

$$TOF = \frac{n_{C_2H_2}}{n_N \times t} \quad (S1)$$

Where n_N is the amount of substance of nitrogen atom in the catalyst, mol; t is the reaction time, s; and n_{C2H2} is the amount of substance of C_2H_2, mol.

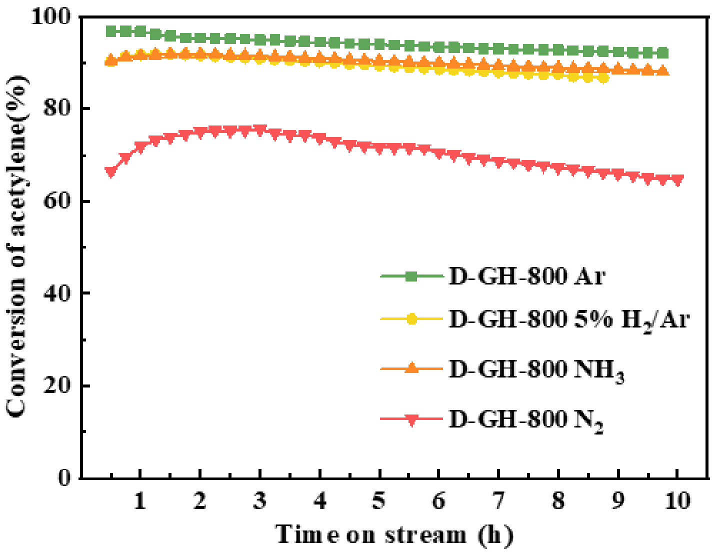

Figure S1. The catalytic performance of catalysts with different calcination atmospheres. Reaction conditions: temperature = 180 °C, C_2H_2 = 36 h^{-1}, velocity V_{HCl}/V_{C2H2} = 1.15.

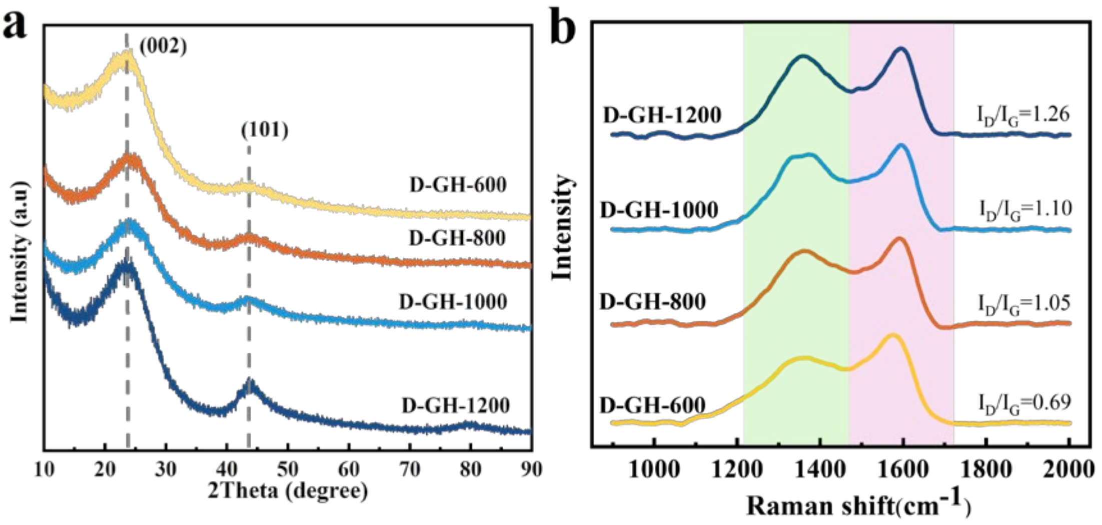

Figure S2. (a) XRD patterns and (b) Raman patterns of D-GH-600, D-GH-800, D-GH-1000 and D-GH-1200.

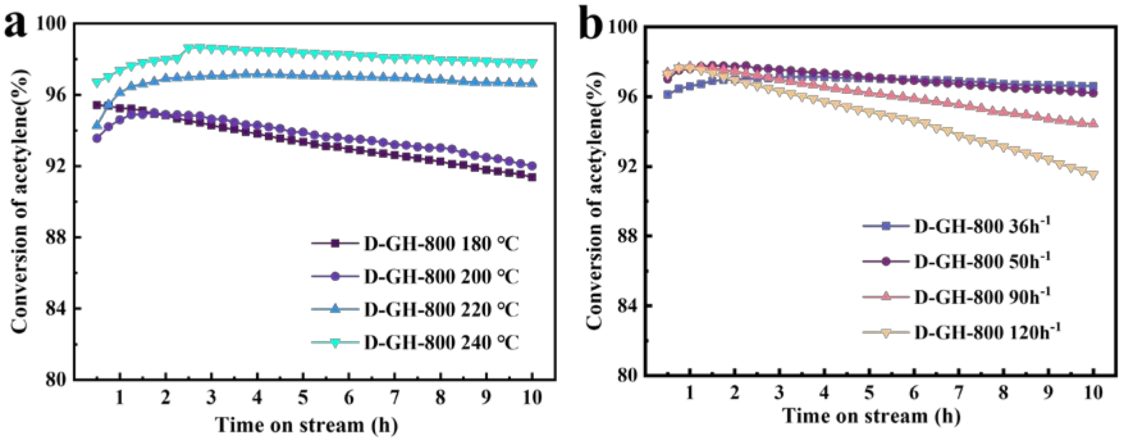

Figure S3. Screening of reaction conditions for catalytic evaluation with D-GH-800 catalyst: (a) reaction temperature. Reaction conditions: temperature = 180 - 240 °C, C_2H_2 = 36 h^{-1}, velocity V_{HCl}/V_{C2H2} = 1.15. (b) reaction airspeed. Reaction conditions: temperature = 220 °C, velocity V_{HCl}/V_{C2H2} = 1.15.

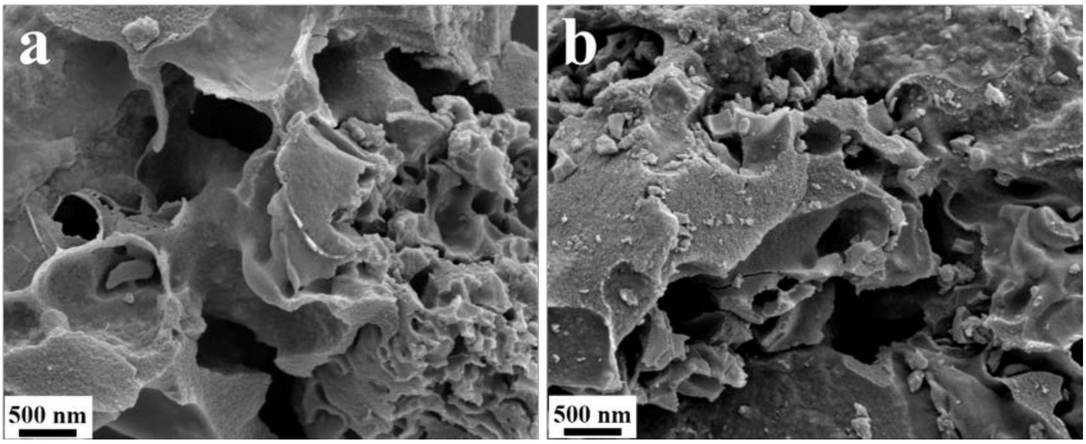

Figure S4 SEM image of D-GH-800 used.

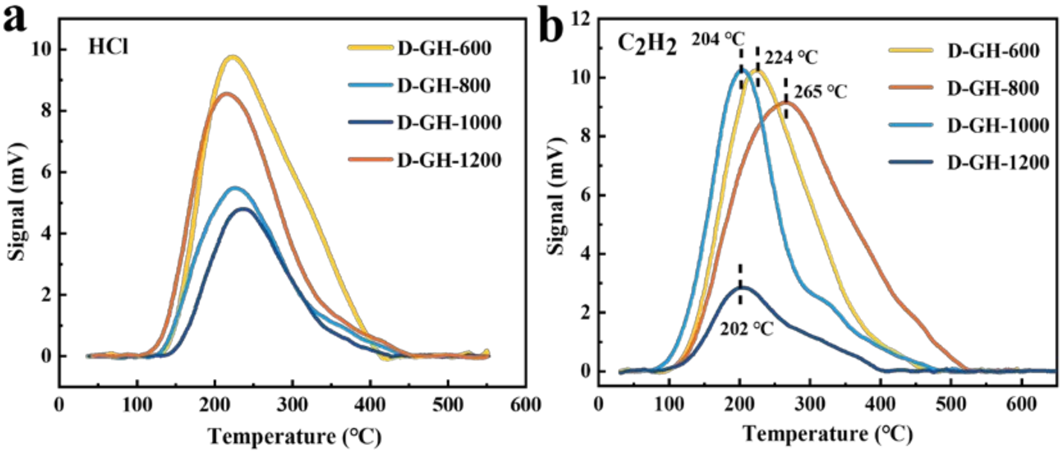

Figure S5. (a) HCl-TPD and (b) C_2H_2-TPD of D-GH catalysts.

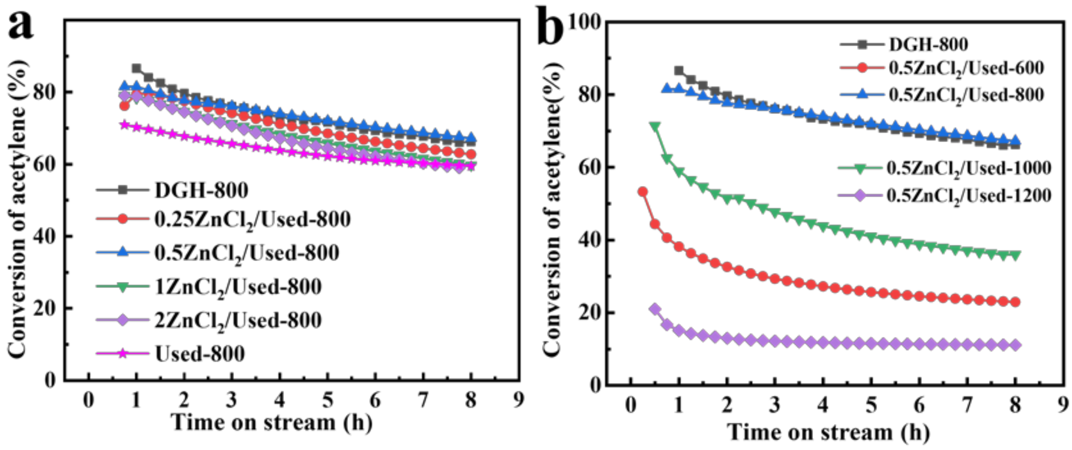

Figure S6. The catalytic performance of (a) x$ZnCl_2$/Used-800 catalysts and (b) 0.5$ZnCl_2$/Used-H catalysts. Reaction conditions: temperature = 180 °C, C_2H_2 = 90 h^{-1}, velocity V_{HCl}/V_{C2H2} = 1.15.

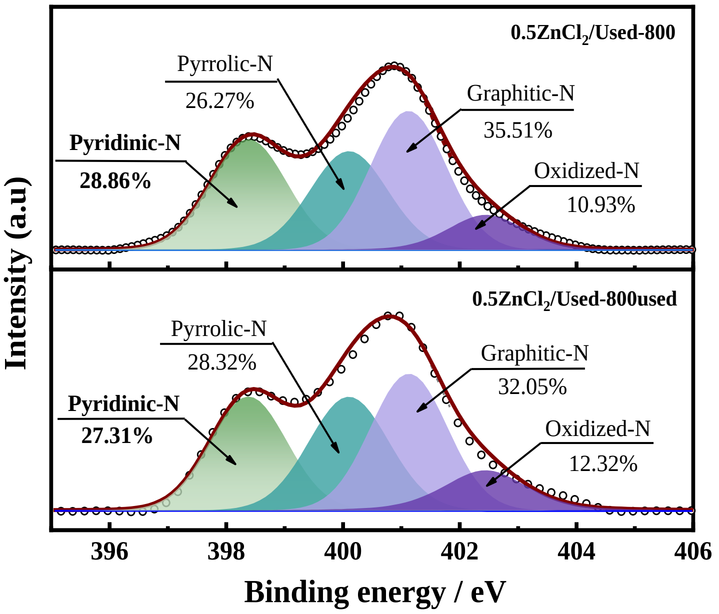

Figure S7 High-resolution XPS N1s spectra for 0.5$ZnCl_2$/Used-800 and 0.5$ZnCl_2$/Used-800used.

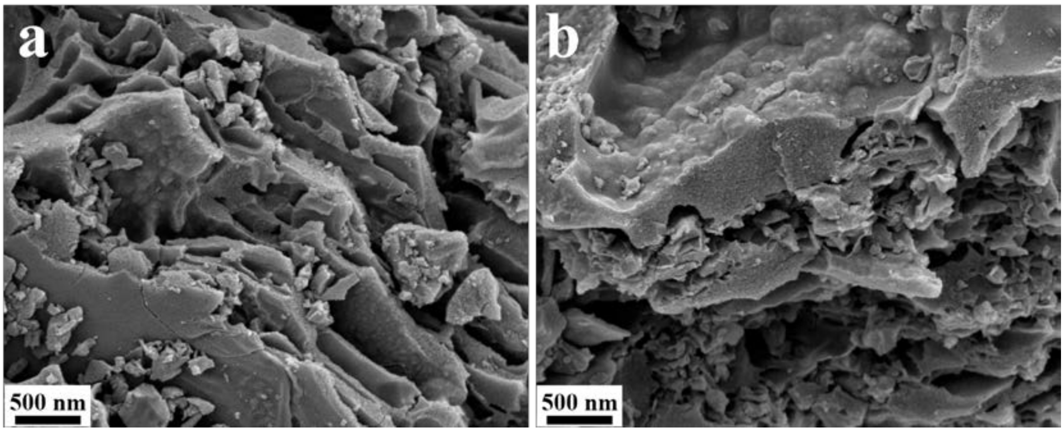

Figure S8. SEM image of $0.5ZnCl_2$/Used-800used catalysts.

Table S1 The TOFs of catalysts based on single nitrogen atoms.

**Sample**	**Nitrogen Content (%)**	**Reaction conditions**	**Acetylene conversion**	**TOF (10−6 s−1)**	**Ref.**
NC-800	13.46	V=2 ml<br>C_2H_2 = 30 h^{-1}	98.06%	4.14	1
SBMC-600	8.90	V=5 ml<br>C_2H_2 = 30 h^{-1}	99.00%	4.63	2
3NR/4CAC	3.40	V=4 ml<br>C_2H_2 = 30 h^{-1}	97.90%	11.25	3
1.00NPC	13.50	V=2 ml<br>C_2H_2 = 30 h^{-1}	87.00%	4.03	9
PACP-800	8..99	V=1 ml<br>C_2H_2 = 30 h^{-1}	86.00%	4.98	10
PoPD-C-800	19.32	V=2 ml<br>C_2H_2 = 200 h^{-1}	98.50%	19.41	11
D-AC-M	1.90	V=5 ml<br>C_2H_2 = 30 h^{-1}	61.60%	16.21	13
NC-800-700	13.55	V=2 ml<br>C_2H_2 = 50 h^{-1}	97.85%	8.36	19
D-GH-800	6.28	V=2 ml<br>C_2H_2 = 50 h^{-1}	99	16.25	This work

Table S2 Content of pyridinic-N, pyrrolic-N, graphitic-N, and Oxidized N in the catalysts.

Catalyst	N	Pyridinic N (398.4 eV)	Pyrrolic N (400.1eV)	Graphitic N (401.1eV)	Oxidized N (402.4eV)
D-GH-600	8.52	33.33	46.24	20.43	-
D-GH-800	6.28	34.64	25.70	29.48	10.18
D-GH-1000	4.08	21.28	24.43	38.71	15.58
D-GH-1200	1.96	10.33	21.44	52.95	15.28

Table S3 Pore structure parameters and element content of various catalysts.

Catalyst	S_{BET} (m^2g^{-1})	V (cm^3g^{-1})	D (nm)	C	N	O	Zn
Used	14.24	0.05	6.35	89.43	6.02	4.55	-
0.5ZnCl₂/Used-800	507.55	0.31	2.72	86.32	6.11	5.57	2.06
0.5ZnCl₂/Used-800used	115.59	0.12	4.05	88.01	5.95	6.04	1.48

References

1. Lu, F.J.; Xu, D.; Lu, Y.S.; Dai, B.; Zhu, M.Y. High nitrogen carbon material with rich defects as a highly efficient metal-free catalyst for excellent catalytic performance of acetylene hydrochlorination. *Chin. J. Chem. Eng.* **2021**, 29, 196–203.
2. Shen, Z.B.; Liu, Y.; Han, Y.J.; Qin, Y.J.; Li, J.H.; Xing, P.; Jiang, B. Nitrogen-doped porous carbon from biomass with superior catalytic performance for acetylene hydrochlorination. *RSC Adv.* **2020**, 10, 14556–14569
3. Mei, S.; Gu, J.J.; Ma, T.Z.; Li, X.Y.; Hu, Y.B.; Li, W.; Zhang, J.L.; Han, Y. N-doped activated carbon from used dyeing wastewater adsorbent as a metal-free catalyst for acetylene hydrochlorination. *Chem. Eng. J.* **2019**, 371, 118–129.
